# Inflexibility of the plasma miRNA response following a high-carbohydrate meal in overweight insulin-resistant women

**DOI:** 10.1186/s12263-020-0660-8

**Published:** 2020-02-04

**Authors:** F. Ramzan, R. F. D’Souza, B. R. Durainayagam, A. M. Milan, N. C. Roy, M. C. Kruger, C. J. Henry, C. J. Mitchell, D. Cameron-Smith

**Affiliations:** 1 The Liggins Institute, The University of Auckland, 85 Park Road, Grafton, Private Bag, 92019, Auckland, 1142 New Zealand; 2 The Riddet Institute, Palmerston North, New Zealand; 3 School of Medical Sciences, The University of Auckland, Auckland, New Zealand; 4 Food Nutrition & Health Team, AgResearch Ltd, Palmerston North, New Zealand; 5 The High-Value Nutrition National Science Challenge, Auckland, New Zealand; 6 Institute of Food Science and Technology, Massey University, Palmerston North, New Zealand; 7 Clinical Nutrition Research Centre, Singapore Institute for Clinical Sciences, Agency for Science, Technology and Research, Singapore, 117609 Singapore; 8 School of Kinesiology, The University of British Columbia, Vancouver, Canada; 9 Food & Bio-Based Products Group, AgResearch Ltd, Palmerston North, New Zealand; 10 Singapore Institute for Clinical Sciences, Agency for Science, Technology and Research (A*STAR), Singapore, 117609 Singapore

**Keywords:** Metabolic inflexibility, Noncoding-RNA, PBMCs, Substrate switching, Gene expression

## Abstract

**Context:**

Metabolic inflexibility is a characteristic of insulin resistance, limiting the ability to transiently regulate oxidative metabolism and gene expression in response to nutrient availability. Little is known of the flexibility of post-transcriptional regulation, including circulatory miRNAs (c-miRNAs).

**Design:**

The abundances of targeted c-miRNAs, with reported functions in metabolic regulation, were analysed in response to a high-carbohydrate meal in healthy weight insulin-sensitive (IS) and overweight insulin-resistant (IR) women.

**Participants:**

Age-matched healthy weight IS (*n* = 20, BMI = 24.3 ± 0.70) and overweight IR (*n* = 20, BMI = 28.6 ± 0.67) women.

**Methods:**

An abundance of c-miRNAs was quantified prior to and following a high-carbohydrate breakfast meal (2500 kJ; 50% carbohydrate, 20% fat and 27% protein). Target genes of the differentially regulated c-miRNA were measured in RNA extracted from circulatory peripheral blood mononuclear cells (PBMCs).

**Results:**

In healthy weight IS women, both miR-15a-5p (*p* = 0.03) and miR-17-5p (*p* < 0.01) levels were halved at 4 h post-meal. These miRNA remained unaltered following the same meal in the overweight IR women. Furthermore, amongst genes targeted by these miRNA, *CPT1A* (*p* = 0.01) and *IL8* (*p* = 0.03) had also reduced expression 4 h post-meal only in the healthy weight IS women.

**Conclusions:**

The study findings provide preliminary evidence for a possible extension of metabolic inflexibility to include c-miRNAs.

**Trial registration:**

The clinical trial is registered with Australian New Zealand Clinical Trials Registry under Trial registration: ANZCTR: ACTRN12615001108505. Registered on 21 October 2015.

## Introduction

Diurnal metabolism involves adaptive tailoring of glucose and lipid oxidation in concert with the physiological demands and nutritional state, thereby precisely meeting whole body energetic demands [1]. Whilst this regulation of nutrient flux is primarily dependent upon enzymatic regulation, dynamic regulation of the transcription of key metabolic genes is also essential [2, 3]. For instance, during fasting, there is an upregulation of sirtuin-3 (*SIRT3*) expression leading to increased fatty acid oxidation by reversible deacetylation of long-chain coenzyme A dehydrogenase (LCAD), a key fatty acid oxidation enzyme [4]. In individuals with cardio-metabolic diseases including obesity and insulin resistance (IR), this capacity to adaptively regulate nutrient fluxes and oxidation to match the physiological and nutritional states is diminished [5, 6], a root cause for advancement of these derangements into serious metabolic diseases including type 2 diabetes mellitus (T2DM) and cardiovascular diseases (CVD) [3]. This loss of flexibility occurs downstream of altered regulation of transcription factors that are in turn controlled by nutrient availability and cellular energy homeostasis [2].

The regulation of metabolic flexibility is reflected at the transcriptional level of gene regulation, yet little is known of possible regulation at the post-transcriptional level, especially by microRNAs (miRNAs). miRNAs are evolutionarily conserved small noncoding RNAs with widespread biological functions [7], mainly acting as negative regulators of post-transcriptional gene expression [8]. Circulatory miRNAs (c-miRNA) are known to play a critical role in cell-to-cell communication [9] and have been increasingly implicated as potential biomarkers of diseases’ state, prognosis and progression, for conditions including T2DM and cardiovascular disease (CVD) (Additional file 1) [10]. Clinical and animal models have further established functional roles of a subset of c-miRNAs, with demonstrated function in regulating the genes involved in multiple aspects of metabolic control and insulin function [11, 12]. Moreover, c-miRNAs are identified to exhibit circadian oscillations [13], and it has been reported that dynamic miRNA-based post-transcriptional regulation of gene expression is important to harmonise physiological transitions during fed-fast-refed cycles [14].

Therefore, the aim of this study was to quantify expression of c-miRNAs with established roles in the regulation of metabolic function and with putative dysregulation in established T2DM (Additional file 1: Table S1), in response to a change in nutrient status from overnight fasted to the postprandial state following a high-carbohydrate meal. The ten selected c-miRNAs for this study were selected based on our previously conducted study reporting c-miRNAs as biomarkers of MetS [15]. This analysis was performed in a selected cohort of post-menopausal women, who were recruited on the basis of metabolic disease risk. On the basis of the in silico functional target analysis of the differentially regulated miRNAs, mRNA was extracted and analysed from the circulatory peripheral blood mononuclear cells (PBMCs). PBMC gene analysis has been reported to be a non-invasive surrogate measure predictive of the molecular mechanisms within tissues which cannot be readily accessed [16]. PBMC gene expression also demonstrates significant concordance (80%) with other tissue types [17]. It was hypothesised that inflexibility in the regulation of c-miRNAs, with established functional roles in nutrient metabolism, would be evident in the IR individuals. Furthermore, the altered c-miRNA responses would correspond with dysregulated expression of genes with known function in regulation of metabolic pathways in PBMCs.

## Methods

### Study design

The study utilised plasma samples from a previously performed randomised controlled cross-over trial, approved by the University of Auckland Human Participants and Ethics Committee (014501). The study was conducted in accordance with the guidelines of Declaration of Helsinki and is registered with the Australian New Zealand Clinical Trials Registry at www.anzctr.org.au (ANZCTR: ACTRN12615001108505). All participants signed the written informed consent.

### Study population and meal

Study participants were categorised into two groups: healthy weight IS (*n* = 20) and overweight IR (*n* = 20). Homeostasis model assessment of insulin resistance (HOMA-IR) was used to estimate insulin sensitivity [18]. Participants with a BMI of > 25 kg/m^2^ and HOMA-IR of ≥ 1.4 were classified as overweight IR, whilst participants with a BMI between 20.0 and 24.9 kg/m^2^ and HOMA-IR < 1.4 were classified as healthy weight IS. Participants with current or past endocrine disorders, CVD, cancer, T2DM or any current medications that might interfere with the study endpoint (e.g., anti-inflammatory drugs) were excluded from the study.

All the participants consumed a standard evening meal, and arrangements were made for them to arrive fasted between 0700 and 0800 h to the Paykel Clinical Research Unit, Liggins Institute. A venous blood sample (EDTA-coated vacutainer) was drawn in the fasted state. The participants then consumed a high-carbohydrate meal breakfast (2500 kJ; 50% carbohydrate, 20% fat and 27% protein) within a 10-min time period (Table 1). All participants consumed the meal in its entirety. Venous blood collection was again performed at 2 and 4 h following meal consumption. Plasma was separated by centrifugation at 1900×*g* for 15 min at 4 °C and was immediately stored at − 80 °C until further analysis.

**Table 1:** Composition of breakfast meal

Breakfast	Serving size	Calories	Protein (g)	Fat (g)	Carbohydrates (g)
Whey protein isolate (WPI) unflavoured (g)	30	116	27.7	0.3	0.15
Anchor butter (g)	12.7	92.3	< 1.0	10.4	< 1.0
Budget white bread (slices)	4	272	11.2	2.4	46.6
Maltodextrin (g)	9	36	0	0	9
Gatorade orange (ml)	300	73.2	0	0	18
Macronutrient composition (%)		116	27.1	20.0	51.3

### Anthropometric and biochemical analysis

Height, weight, waist circumference and blood pressure were measured at fasting. Both fasting and postprandial plasma glucose and triglycerides were measured using Cobas Modular P800 (Roche Diagnostics, New Zealand). Plasma insulin fasting and postprandial was measured using a Cobas Modular E170 analyser (Roche Diagnostics, New Zealand). Homeostasis model assessment of insulin resistance (HOMA-IR) was calculated to estimate insulin sensitivity using the equation by Matthews et al. [18]. An insulinogenic index (ΔInsulin_30_/ΔGlucose_30_ ratio) was calculated to assess early insulin secretion in response to the meal [19]. The area under the curve (AUC) for measurement of insulin and triglyceride concentrations at baseline and at 2 and 4 h post-meal was calculated.

### Circulating total RNA extraction

Briefly, 250 μl plasma was used for total RNA extraction (including miRNAs) using a previously described by D’Souza et al. [20]. A fixed volume of plasma was utilised to minimise extraction variation between different samples and time points [21].

### cDNA synthesis and circulating miRNA quantitative PCR (qPCR)

Two microlitres of total RNA was used as an input for cDNA synthesis reaction using TaqMan™ Advanced miRNA cDNA Synthesis Kit (Catalogue number: A28007, Applied Biosystems, USA), according to the manufacturer’s recommendations. For quantification of circulatory miRNA abundances using qPCR analysis, custom human miRNA assays of miR-15a-5p,-miR-16-5p, miR-17-5p, miR-21-3p, miR-126-3p and miR-222-3p were used (TaqMan MicroRNA Assays, Applied Biosystems, USA). Quantification was performed on a Quant Studio™ 6 Flex Real-Time PCR System (Thermo Fisher Scientific, USA). Samples with a detected cycle threshold (Ct) of ≤ 35 were included in the analysis.

For normalisation of expression data, a geometric mean of an endogenous miRNA (miR-423-5p) and an exogenous spike-in (cel-miR-238) used for quality control were performed [22]. Haemolysis of all samples was monitored by comparing miR-451a expression (a highly expressed miRNA in red blood cells) with miR-23a-3p expression (a miRNA unaffected by haemolysis) [23]. The resulting ΔCt (miR-23a-3p–miR-451a) was used as a measure of the degree of haemolysis; two samples with a ΔCt of > 7 were excluded from further analysis. The abundance of miRNAs was measured using the two (−ΔCt) method [24].

### In silico target analysis

Target gene prediction network analysis of the differentially expressed miRNAs and over-representation analysis of the targeted genes were performed using miRNet [25]. All set of genes targeted by the miRNAs were identified and were subsequently used for prediction of targeted pathways by these miRNAs. Functional annotation of the dysregulated miRNA and the identification of miRNA-target gene controlled pathways were determined via Gene Ontology (GO) categories biological process analysis based on the hypergeometric tests with *p* values ≤ 0.05 adjusted for false discovery rate (FDR).

### Peripheral blood mononuclear cells (PBMC) total RNA extraction

Total RNA was isolated from approximately 2.5 × 10^6^ PBMCs collected at fasting as well at 4 h post-meal using the AllPrep® DNA/RNA/miRNA Universal Kit (QIAGEN, Germany) following the manufacturer’s protocol [26].

### qPCR gene expression analysis

Input RNA of 500 ng was used for cDNA synthesis using the High Capacity RNA-to-cDNA™ kit (Life Technologies, USA). Quantification of gene expression (mRNA) was performed by qPCR on a LightCycler 480 II (Roche Applied Science, Germany) using LightCycler® 480 SYBR Green I Master (Roche Applied Science, Germany). Genes quantified included peroxisome proliferator-activated receptor (*PPARA*), carnitine palmitoyltransferase-1A (*CPT1A*), acyl-CoA oxidase-1 (*ACOX1*), *CD36*, *USP3*, mitofusion-2 (*MFN2*), *SMAD3*, vascular endothelial growth factor-A (*VEGFA*) and pro-inflammatory cytokines (interleukin-6 (*IL6*), tumour necrosis factor-alpha (*TNF-α*) and interleukin-8 (*IL8*)). Primers for qPCR were designed using BLAST software (Additional file 1: Table S2) [27]. For normalisation of the PCR data, the geometric mean [28] of three human reference genes [29, 30], valosin-containing protein (*VCP*), charged multivesicular body protein 2A (*CHMP2A*) and chromosome 1 open reading frame 43 (*C1orf43*), were used. Primer efficiency for every target was calculated using the slope of standard curve, and only primers with an efficiency of 90–100% were used for analysis [31]. The relative expression of mRNA was measured using the 2(^ΔCt^) method [24].

### Statistical analysis

The expression data were evaluated for normality using the Shapiro-Wilk test. The differences in the abundance of c-miRNA, PBMC genes, AUC _insulin_ and AUC_TG_ in relation to the acute dose of meal were measured using repeated-measures ANOVA, with time as a repeated factor and group as a between-subject factor, followed by Holm-Sidak multiple comparison corrections. Samples with an expression of more than three times the interquartile range were treated as outliers and were subsequently removed from further analysis [32]. Data are shown as means ± SD unless otherwise stated. Analyses were carried out using SPSS version 25.0 (SPSS Inc., USA) and graphs constructed using GraphPad prism-7 (GraphPad Software, USA). Statistical significance was set at *p* ≤ 0.05.

## Results

### Study population characteristics

Participant clinical and demographic characteristics are summarised in Table 2. Study participants in both the healthy weight IS (*n* = 20) and overweight IR (*n* = 20) group did not differ in terms of age.

**Table 2:** Characteristics of study participants

	Healthy weight IS (*n* = 20)	Overweight IR (*n* = 20)	*p* value
Age (years)	63.5 ± 1.0	62.5 ± 1.3	0.57
Weight (kg)	62.3 ± 2.1	77.6 ± 2.1	< 0.001
Height (cm)	159.9 ± 1.4	164.5 ± 1.2	0.01
BMI (kg/m^2^)	24.3 ± 0.7	28.7 ± 0.7	< 0.001
Waist circumference (cm)	80.4 ± 2.2	93.8 ± 1.7	< 0.001
Systolic blood pressure (mmHg)	121.6 ± 3.0	139.2 ± 3.0	< 0.001
Diastolic blood pressure (mmHg)	67.0 ± 2.2	72.0 ± 2.3	0.09
Plasma glucose (mg)	99.3 ± 1.8	108.6 ± 1.8	0.57
HDLc (mM)	2.1 ± 0.1	1.7 ± 0.1	< 0.01
Triglycerides (mM)	0.91 ± 0.1	1.40 ± 0.1	< 0.01
Insulin (mIU)	5.2 ± 0.5	9.1 ± 0.8	0.01
Medication use (*n*)			
Aspirin	2	1	
Statins	0	3	
ACE inhibitor	0	3	
α-Blocker	0	1	
β-Blocker	0	1	
PPIs	0	1	
SSRIs	1	2	

Values are means ± SD

*HDLc* high-density lipoprotein cholesterol, *ACE* angiotensin-converting enzyme inhibitor, *PPIs* proton pump inhibitors, *SSRI* selective serotonin reuptake inhibitors

### Biochemical measures

**Fig. 1:**
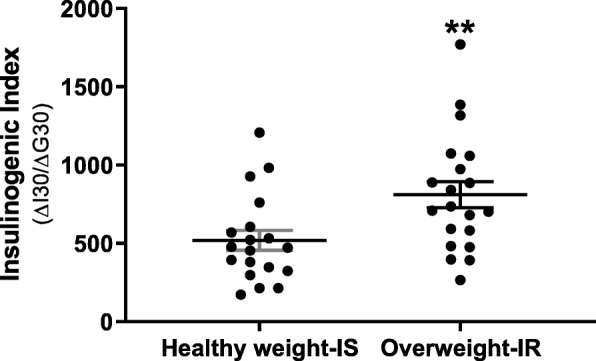
Insulinogenic index (△I30/△G30). Estimated after a high-carbohydrate meal in healthy weight IS and overweight IR women, Black dots represent individual study subjects. An asterisk indicates a significant difference in the insulinogenic index of overweight IR as compared to healthy weight IS women (two asterisks denote *p* ≤ 0.01, error bars represent the standard error of mean (SEM))

**Fig. 2:**
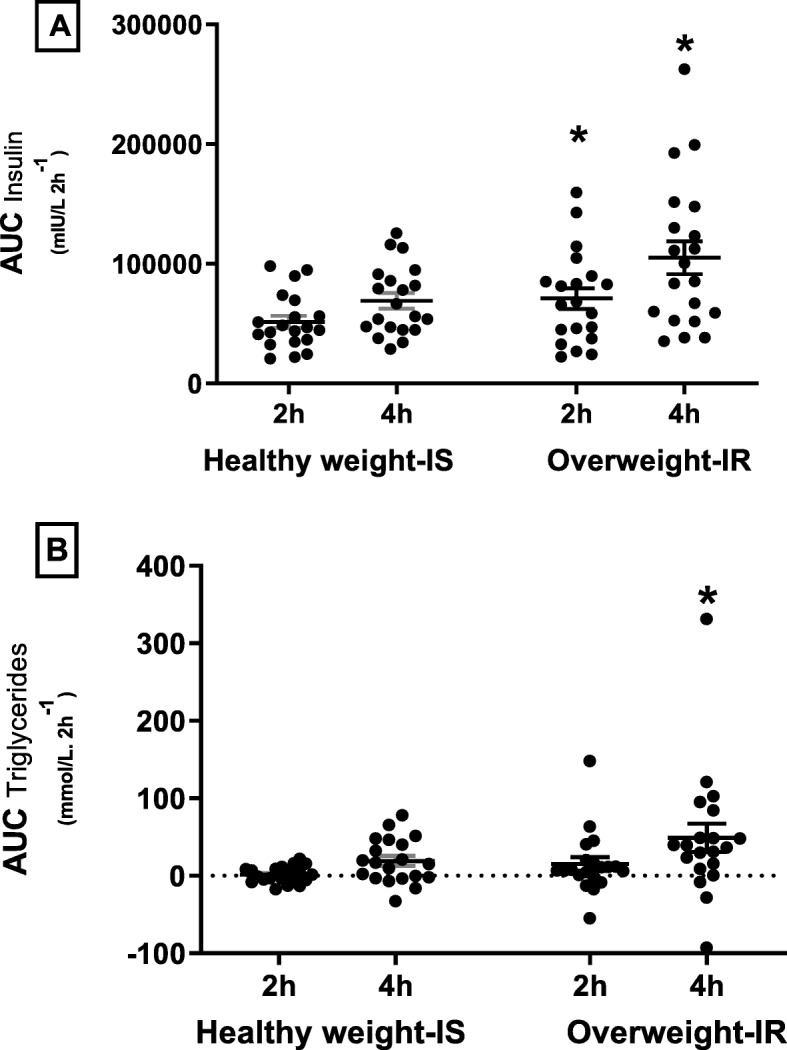
Area under the curve (AUC). **a** AUC for Insulin. **b** AUC for triglycerides. Black dots represent study individual study subjects; An asterisk denotes between the group difference at an indicated time point (*p* ≤ 0.05); error bars represent the standard error of mean (SEM)

Overweight IR as compared to healthy weight IS women had a greater insulinogenic index (mIU/L/mg/dL) (810.9 ± 84.1 versus 518.8 ± 63.8, respectively; *p* < 0.01; Fig. 1) following the meal. Mean AUC insulin (mIU/L 120 min^−1^) was higher in overweight IR subjects as compared to healthy weight IS (58,749.7 ± 5179.4 and 40,207.2 ± 5179.4, respectively; *p* ≤ 0.05; Fig. 2a). In addition, mean AUC triglycerides (TG; mmol/L 120 min^−1^) was higher in overweight IR women as compared to healthy weight IS (21.7 ± 7.2 and 7.1 ± 2.5, respectively; *p* ≤ 0.05; Fig. 2b).

### Postprandial abundance of circulatory miRNAs

**Fig. 3:**
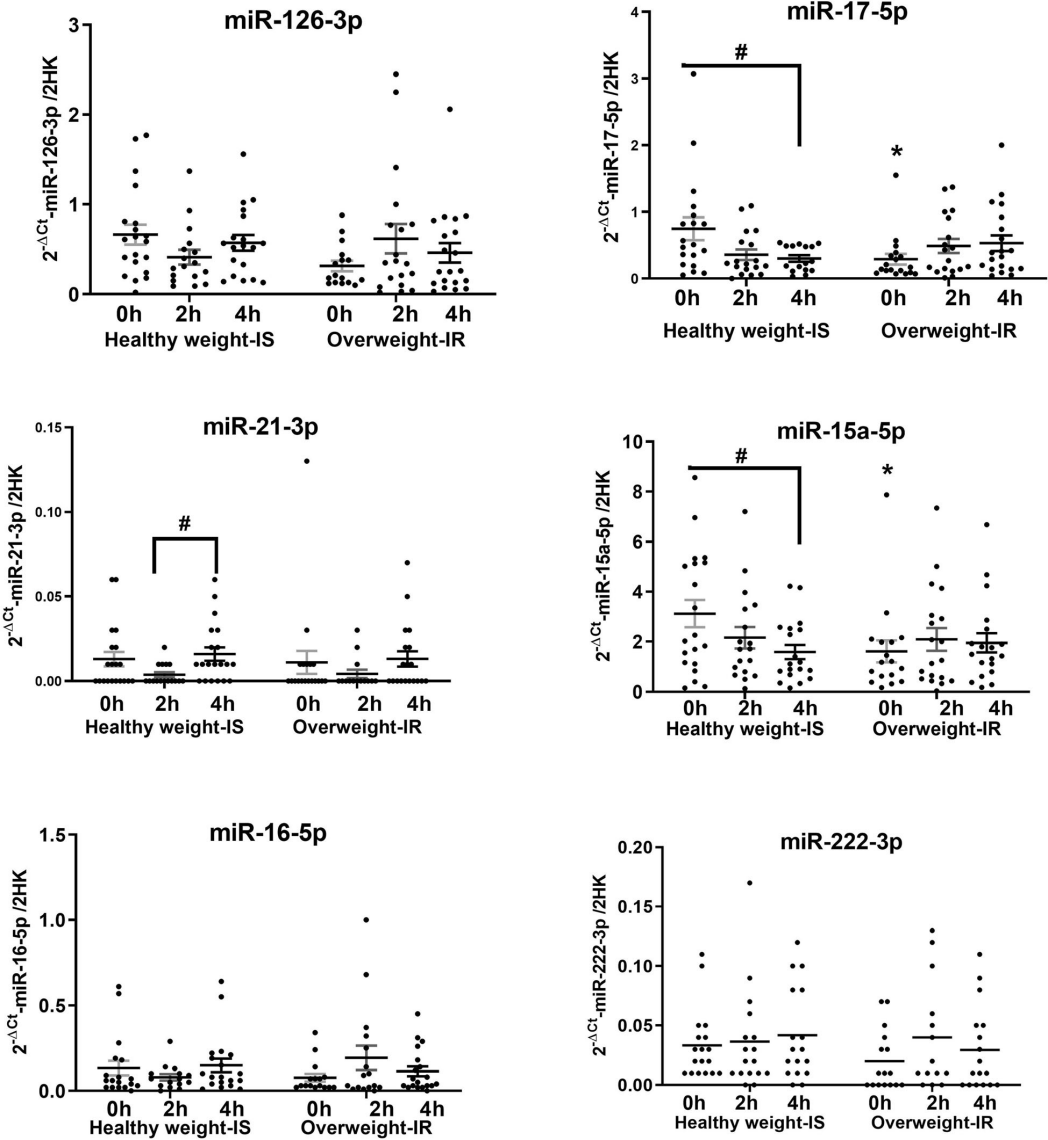
Differential expression of circulatory miRNAs at baseline and in response to single meal at 2 h and 4 h. Black dots represent study subjects, GxT denotes group and time interactions and an asterisk denotes a statistically significant difference between the two groups at indicated time point (*p* ≤ 0.05). Number sign denotes a statistically significant difference within the group at indicated time points in relation to baseline *(*^#^*p* ≤ 0.05; ^##^*p* ≤ 0.01). Black lines indicate statistically significant differences between different time points within the group

Differences in the fasting miRNA expression in this cohort have been previously reported in [15]. Comparison of the abundance of circulatory miRNAs at fasting and postprandially 2 and 4 h in overweight IR subjects (*n* = 20) to those of healthy weight IS (*n* = 20) demonstrated differences in the circulating levels of miR-15a-5p and miR-17-5p. The postprandial responses in the levels of these miRNAs diverged between the two groups (group × time interaction; miR-15a-5p (*p* < 0.01) and miR-17-5p (*p* = 0.01)) (Fig. 3). In healthy weight IS women, miR-15a-5p (*p* = 0.03) and miR-17-5p (*p* < 0.01) exhibited halving of abundances following the meal. In contrast, overweight IR subjects showed no significant change in the abundance of these miRNAs during the postprandial period (2 or 4 h).

### Prediction of downstream mRNAs

**Fig. 4:**
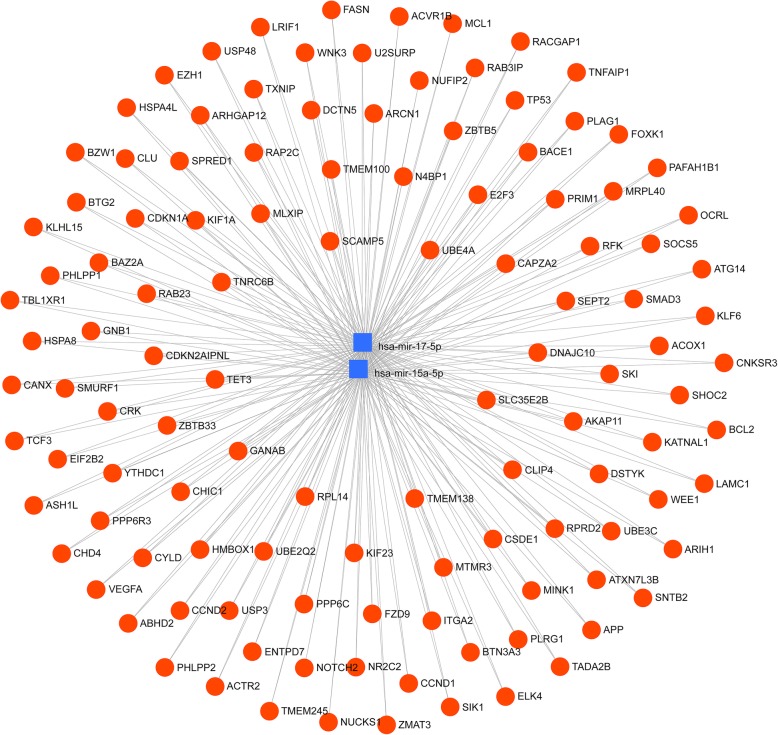
Network gene analysis of the differentially expressed miRNA. The network visualisation of the differentially expressed miRNAs and their respective gene targets; blue squares represent miRNAs; red circles represent shared genes between the miRNAs

Target gene prediction analysis demonstrated 1781 genes (both strong and weak interaction) as being putatively regulated by miR-15a-5p and miR-17-5p, with 117 of these targets strongly being regulated by both of these miRNAs (Fig. 4). Over-representation analysis of all the targeted genes identified 175 computed GO biological processes significantly enriched by the target genes of these miRNAs (Additional file 1: Table S3) [33].

Functional analysis of the shared targeted genes (117) highlighted 26 of the GO categories being significantly enriched by these miRNAs. Amongst these categories, 14 processes predominantly involved in the regulation of cellular and macromolecular metabolism were enriched (*p* ≤ 0.05) (Additional file 1: Table S4) [33]. Modifications in these pathways have been previously described as associated with the regulation of metabolic homeostasis [34–36]. Interestingly, of the top 50 shared genes, 5 genes (ACOX1, *USP3*, *SMAD3*, *VEGFA* and *CD36*) were found to be uniformly enriched in almost all of the identified metabolic processes; therefore, these genes were further quantified in PBMCs using qPCR. Along with these shared targets, additional genes *CPT1A*, *MNF2* and *PPARA* [2, 37] and pro-inflammatory cytokines *(TNF-α*, *IL6* and *IL8*) [3] which were reported to be targeted by either miR-15a-5p or miR-17-5p from our in silico analysis as well as were found to be involved in lipid and oxidative metabolism based on our literature search with a criteria of being reported in at least two of the models amongst animals, humans or cell lines were shortlisted for PBMC quantification.

### PBMC gene expression

**Fig. 5:**
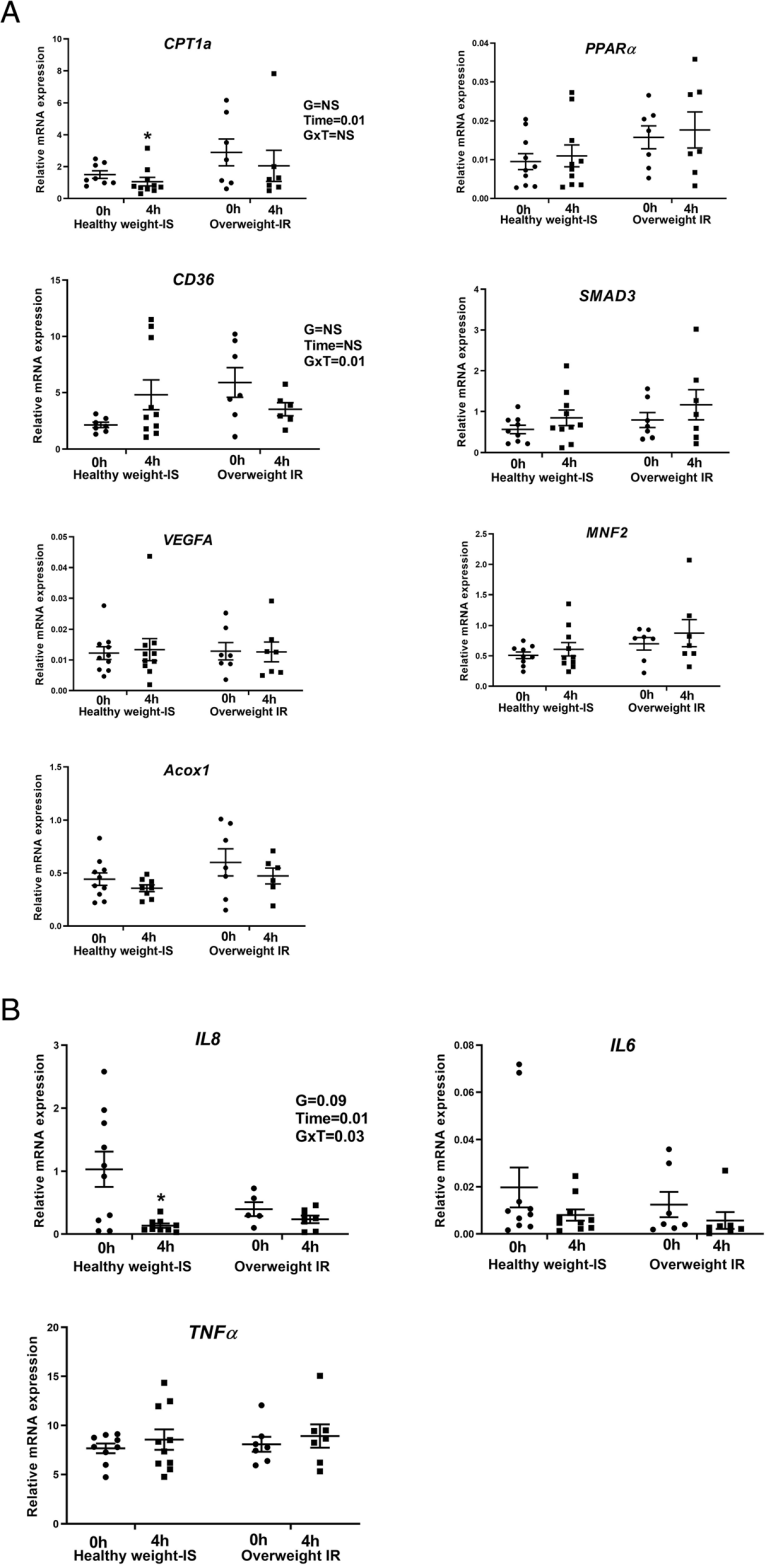
Quantification of PBMC gene expression involved in regulating fuel metabolism and inflammatory-related pathways at baseline and in response to single meal at 4 h. **a** Differential expression of metabolic genes at baseline and in response to single meal at 4 h. **b** Differential expression of pro-inflammatory cytokines at baseline and in response to single meal at 4 h. Black dots represent individual study subjects. GXT denotes group and time interactions. An asterisk denotes a statistically significant difference between the two groups at an indicated time point (*p* ≤ 0.05). An asterisk indicates *p* ≤ 0.05, and two asterisks indicate *p* ≤ 0.01

No difference in expression levels of measured PBMC mRNA was observed between the groups at fasting. A decrease in the expression of *CPT1A* (*p* = 0.01) (Fig. 5a) was observed in the healthy weight IS women at 4 h following the meal. Furthermore, there was a group × time interaction (*p* = 0.03) for expression of *IL8*, where normal weight IS women displayed a significant reduction in expression of *IL8*, 4 h post-meal (*p* = 0.01) (Fig. 5b). No changes in the gene expression of *PPARA*, *SMAD3*, *VEGFA*, *MFN2*, *CD36*, *ACOX1*, *IL6* and *TNF-α* were observed either between the groups or following the meal. Although *USP3* was also a claimed candidate, its expression was too lowly expressed to be identified in the current sample set*.*

## Discussion

Metabolic flexibility is a hallmark feature of metabolic health and insulin sensitivity [1]. The loss of the ability to precisely tailor and regulate metabolic fluxes is a major component in the metabolic dysregulation experienced in IR states, but may also be a key feature in the progression towards serious disease pathologies, as experienced in T2DM and CVD [5, 38]. In this study, the abundances of selected c-miRNAs, with established interaction with gene pathways necessary for nutrient homeostatic regulation, were analysed in response to a high-carbohydrate breakfast meal. Significant differences in the postprandial responsiveness of c-miR-15a-5p and c-miR-17-5p were observed. In the overweight IR subjects, these c-miRNAs had reduced fasting abundances, which remained unaltered within 4 h of the high-carbohydrate meal. In the healthy weight IS, both c-miR-15a-5p and c-miR-17-5p abundances declined ~ 50% in the same time period.

miR-15a-5p and miR-17-5p are implicated in a variety of experimental circumstances to influence metabolic function and insulin sensitivity [39, 40]. miR-15a-5p is primarily secreted into the circulation from pancreatic β-cells [41] and is reported to influence pancreatic differentiation and development and promote glucose-stimulated insulin secretion and biosynthesis [42]. Evidence has shown reductions in the abundance of c-miR-15a-5p in morbidly obese men [43] and type 2 diabetics [44]. Consistent with these findings, we have previously demonstrated a reduction in its expression at fasting [15]. Whilst further demonstrating reduced circulating abundance in only healthy weight IS women following the meal, this current study might suggest the loss of responsiveness of miR-15a-5p to altered nutrient status and therefore could be responsible for the inflexibility in the metabolism of subjects who are at a higher risk of developing associated chronic metabolic diseases.

miR-17-5p has also reported to be central to the proliferation and adaptation of pancreatic β-cells [45]. miR-17/92 family is also reported to be involved in promoting adipocyte differentiation, with their dysregulation leading to the development of adipose-related vascular diseases [46, 47]. However, little is known about the circulatory abundance of miR-17-5p in obesity, with only one study previously identified a reduced abundance of c-miR-17-5p in obese patients [48]. Therefore, the present study provides additional evidence that reduced c-miR-17-5p might be a contributory factor in the development of metabolic inflexibility in states of elevated body fatness.

Although no previous human studies can be identified that have addressed the regulation of c-miR-15a-5p and miR-17-5p in response to meals or dietary manipulation, both miRNAs are reported to have putative roles in regulating genes involved in the coordination of nutrient flux, including fatty acid synthase (*FASN*) [49, 50] and peroxisome proliferator-activated receptor (*PPARA*) [51, 52]. Therefore, to ascertain the effect of observed inflexibility in the expression of miR-15a-5p and miR-17-5p on the regulation of metabolic genes, the current study further analysed the circulating PBMC target gene expression of miR-15a-5p and miR-17-5p reported to be involved in lipid as well as oxidative metabolism as highlighted by the literature search [2, 37] and further supported by in silico analysis [33]. For the majority of genes (*PPARA*, *ACOX1*, *CD36*, *MFN2*, *SMAD3*, *VEGFA*, *USP3*) and pro-inflammatory cytokines *(IL6* and *TNF-α*) analysed, there was no evidence of altered expression either between the healthy weight IS or overweight IR groups in the overnight fasted state or in response to the meal. However, there was an impaired suppression of *CPT1A* in the overweight IR women in response to the meal.

Dynamic regulation of *CPT1A* expression is observed in rodents in the transition from fasted to the fed state [53]. Moreover, miR-17-5p is reported to control the transcription of *CPT1A* gene, mediated through its impact on *PPARA* expression [54]. Recent evidence demonstrates that both miR-15a-5p and miR-17-5p are part of a coordinated network of nutrient-sensitive miRNA in mouse liver [53], with loss of dynamic regulation of the hepatic miRNA network resulting in accelerated gluconeogenesis and failed catabolic-to-anabolic switching upon feeding in these mice. Taking into account the important role of *CPT1A* and miRNA networks in the regulation of metabolic homeostasis, the current study suggests a possible link between meal-induced *CPT1A* gene expression and miRNA regulation.

Both miR-15a-5p and miR-17-5p are also reported to be involved in the regulation of inflammation, through a specific targeting of the *IL8* gene [55, 56]. This study also demonstrated a ninefold reduction in the expression of *IL8*, a pro-inflammatory cytokine in the healthy weight IS women, but not in overweight IR women, 4 h after the meal. Little is known of the transcriptional regulation of *IL*8 to altered nutrient availability*.* Evidence shows increased circulating concentrations of *IL8* protein in obesity and diabetes [57]. As the current study did not measure circulating abundances of cytokines, the significance of this measured gene change within the PBMC cell population was not established.

### Limitations

There are several limitations to consider in this current study. Although oxidative metabolism is reported to be inflexible in the overweight IR states, this was not measured in the present study. Such analysis would typically require indirect calorimetry to determine the substrate utilisation as measured by the respiratory quotient (RQ) [58]. However, given that metabolic inflexibility is a common feature of insulin resistance, it is likely that the participants of the current study did experience some impairment in carbohydrate oxidation after the meal. With respect to the analysis of c-miRNA, both sexual dimorphism and ethnicity have a significant bearing on the circulating abundances of many c-miRNA species [59, 60]. As this study was conducted only in Caucasian women, the conclusions may not be translatable to either males or individuals of differing ethnicities. This study also undertook only limited and targeted PCR-based analysis of both c-miRNA and mRNA, with the latter performed only in circulatory PBMC cells. Although PBMCs have been widely used as surrogate tissue to understand whole-body metabolic status, they are not always an appropriate surrogate [61]. Lastly, there is no widely accepted minimal threshold for miRNA abundance profiling [62], which is not always an appropriate surrogate consideration when aiming to differentiate between the biological significance of experimental noise. Thus it remains making it difficult to interpret the biological importance of small changes as observed in this study. Therefore, any subsequent analysis would be improved with the adoption of high-throughput sequencing strategies [26] and larger population cohorts to more comprehensively evaluate the biological significance of global regulation of noncoding and coding RNA transcripts.

## Conclusion

In conclusion, c-miR-15a-5p and c-miR-17-5p failed to respond to a high-carbohydrate meal in individuals with IR, this might be indicative of the inflexibility in the regulation of miRNA to adaptively regulate nutrient flux to respond to the changing nutritional status and energetic demands. Further, whilst it was also demonstrated that the *CPT1A* and *IL8* gene expressions altered post-meal when analysed from a PBMC population, it is unclear whether this is evident either of transcriptional inflexibility or related to the inflexibility in miR-15a-5p and miR-17-5p. However, it is apparent from this data that dysregulated c-miR-15a-5p and c-miR-17-5p to changing nutrient status could be another molecular feature of the metabolic inflexibility that is important in the widespread loss of metabolic control and disease pathobiology of insulin-resistant states. This study data therefore further suggest as yet poorly understood role for c-miRNA in the adaptive regulation of whole-body responsiveness to altered nutritional status. This and many additional studies demonstrate the possible value of c-miRNAs as minimally invasive biomarkers of disease risk, diagnosis and progression [10]. This study further suggests possible use to examine dynamic and diurnal changes as yet another tool to more precisely identify disease risk.

## Supplementary information

**Additional file 1: Table S1.** Circulatory miRNA identified in multiple studies to be important in the regulation of key aspects of cardiometabolic diseases. **Table S2.** Primer sequences of analysed genes. **Table S3.** Overrepresentation analysis of genes targeted by differentially expressed miRNAs (miR-15a-5p, and -17-5p) identifies significant enrichment in Gene Ontology (GO). **Table S4.** Overrepresentation analysis of shared genes between the differentially expressed miRNAs highlights target genes involved in metabolic related pathways.

## Data Availability

The data generated and analysed during this study are available from the corresponding author on reasonable request.
