# Supplementary material for: Inflexibility of the plasma miRNA response following a high-carbohydrate meal in overweight insulin-resistant women
Source: Genes Nutr. 2020 Feb 4;15:2. doi: 10.1186/s12263-020-0660-8 (PMC7001289; doi:10.1186/s12263-020-0660-8)
Supplement: Supplementary file 1 — Additional file 1: Table S1. Circulatory miRNA identified in multiple studies to be important in the regulation of key aspects of cardiometabolic diseases. Table S2. Primer sequences of analysed genes. Table S3. Overrepresentation analysis of genes targeted by differentially expressed miRNAs (miR-15a-5p, and -17-5p) identifies significant enrichment in Gene Ontology (GO). Table S4. Overrepresentation analysis of shared genes between the differentially expressed miRNAs highlights target genes involved in metabolic related pathways. [file 12263_2020_660_MOESM1_ESM.docx]

**Table S1:** Circulatory miRNA identified in multiple studies to be important in the regulation of key aspects of cardiometabolic diseases

|  | miRNAs | Expression | Experimental model | Potential Targets | Disease | Ref. |
| --- | --- | --- | --- | --- | --- | --- |
| 1 | miR-15a-5p | Downregulated | Human Plasma | Angiogenesis/B-cell function | Atherosclerosis/Obesity | [1,2][3,4] |
| 2 | miR-16 | Downregulated | Cell culture | Inflammation | Atherosclerosis | [5][6] |
| 3 | miR-17-5p | Downregulated | Mice | Glucose metabolism/  inflammation | Obesity/  T2DM | [7][8] |
| 4 | miR-21 | Downregulated  Upregulated  Upregulated | Human Plasma  PBMCs  Human Plasma | Lipid Metabolism  Inflammation  (Treg Cells)  Apoptosis/  proliferation | NAFLD  CHD  CHD with T2DM | [9]  [10][11]  [12] |
| 5 | miR-126 | Downregulated | Mice/ Human Plasma | Inflammation | Atherosclerosis/ T2DM | [13]  [14][15]  [16][17] |
| 6 | miR-222 | Downregulated  Upregulated | Mouse  Human Plasma/  Amniotic Fluid | Beta Cell proliferation  Adipogenesis | Insulin deficient  Obesity | [18]  [19] |
| 7 | miR-320a | Upregulation | Human plasma | Insulin Pathway | T2DM/obesity | [20][21][22] |
| 8 | miR-370-3p | Upregulated | Human Plasma | Lipid metabolism | T2DM/Diabetic cardiomyopathy | [23–25] |
| 9 | miR-375 | Upregulated | Human Serum | Beta cell apoptosis | T2DM | [26][27] |
| 10 | miR-451a | Downregulated | Human Liver/serum/Cell culture/Rat | Inflammation/glucose homeostasis | NAFLD/T2DM | [28–30] |

**Table S2**: Primer sequences of analysed genes

| Gene | Forward Primer Sequence | Reverse Primer Sequence |
| --- | --- | --- |
| *VCP* | AAACTCATGGCGAGGTGGAG | TGTCAAAGCGACCAAATCGC |
| *EMC7* | GGGCTGGACAGACTTTCTAATG | CTCCATTTCCCGTCTCATGTCAG |
| *CHMP2A* | CGCTATGTGCGCAAGTTTGT | GGGGCAACTTCAGCTGTCTG |
| *C1orf43* | CTATGGGACAGGGGTCTTTGG | TTTGGCTGCTGACTGGTGAT |
| *VEGF* | TCTTCAAGCCATCCTGTGT | CTTTCTTTGGTCTGCATTC |
| *PPAR-α* | CGCGGCCCAGGCTGAA | ACCAGATGGTGCTGGTTGTG |
| *CPT1a* | TTTGGACCGGTTGCTGATGAC | GCTTCTTTCAGGTGCCTTCC |
| *Mfn-2* | TGATGCAGACGGAAAAGCACT | GTACAACGCTCCATGTGCTG |
| *CD36* | GGTAAAAGGAATCTGTCCTATTGGG | AAAGGTGGAAATGAGGCTGC |
| *mTOR* | GAATTGGCACAGGTTAGTGGC | CAACTGTCCCAGGGTCCAC |
| *ACOX-1* | TATGCCCAGACAGAGATGGGT | TGAAGTCTTTCCAAGCCCAC |
| *SMAD-3* | ACCACCAGATGAACCACAGC | TAACTGGCTGCAGGTCCAAG |
| *TNF-α* | AGCCCATGTTGTAGCAAACC | TGAGGTACAGGCCCTCTGAT |
| *IL-6* | TCAATGAGGAGACTTGCCTGG | GGGTCAGGGGTGGTTATTGC |
| *IL-8* | ACCGGAAGGAACCATCTCAC | GGCAAAACTGCACCTTCACAC |

**Table S3**: Overrepresentation analysis of genes targeted by differentially expressed miRNAs (miR-15a-5p, and -17-5p) identifies significant enrichment in Gene Ontology (GO)

| **GO categories** | **Pval** |
| --- | --- |
| Regulation of cellular protein metabolic process | 0.00 |
| Regulation of cell cycle | 0.00 |
| Negative regulation of transcription from RNA polymerase II promoter | 0.00 |
| Regulation of translation | 0.00 |
| Histone modification | 0.00 |
| Epidermal growth factor receptor signaling pathway | 0.00 |
| Interphase of mitotic cell cycle | 0.00 |
| Covalent chromatin modification | 0.00 |
| Negative regulation of cell cycle | 0.00 |
| Interphase | 0.00 |
| Cellular macromolecule catabolic process | 0.00 |
| DNA-dependent transcription, initiation | 0.00 |
| Cell cycle arrest | 0.00 |
| Regulation of transcription from RNA polymerase II promoter | 0.00 |
| Transcription initiation from RNA polymerase II promoter | 0.00 |
| Regulation of transforming growth factor beta receptor signaling pathway | 0.00 |
| Negative regulation of cellular protein metabolic process | 0.00 |
| Chromatin modification | 0.00 |
| Regulation of protein modification process | 0.00 |
| Cellular protein catabolic process | 0.00 |
| Nuclear import | 0.00 |
| G1 phase of mitotic cell cycle | 0.00 |
| Protein ubiquitination | 0.00 |
| Protein import into nucleus | 0.00 |
| Positive regulation of transcription from RNA polymerase II promoter | 0.00 |
| Viral reproductive process | 0.00 |
| Regulation of protein stability | 0.00 |
| G1/S transition of mitotic cell cycle | 0.00 |
| G1 phase | 0.00 |
| Cell division | 0.00 |
| Intrinsic apoptotic signaling pathway | 0.00 |
| Transforming growth factor beta receptor signaling pathway | 0.00 |
| Positive regulation of cellular protein metabolic process | 0.00 |
| Negative regulation of transcription, DNA-dependent | 0.00 |
| Negative regulation of transcription, DNA-dependent | 0.00 |
| Negative regulation of protein metabolic process | 0.00 |
| Apoptotic signaling pathway | 0.00 |
| Vesicle localization | 0.00 |
| Protein import | 0.00 |
| Regulation of phosphorylation | 0.00 |
| Negative regulation of RNA metabolic process | 0.00 |
| Chromatin organization | 0.00 |
| DNA damage checkpoint | 0.00 |
| Macromolecule catabolic process | 0.00 |
| Macromolecule catabolic process | 0.00 |
| Regulation of protein phosphorylation | 0.00 |
| Negative regulation of cell proliferation | 0.00 |
| Regulation of kinase activity | 0.00 |
| Regulation of protein metabolic process | 0.00 |
| Positive regulation of transcription, DNA-dependent | 0.00 |
| Cell cycle | 0.00 |
| Positive regulation of transcription, DNA-dependent | 0.00 |
| Protein catabolic process | 0.00 |
| Protein localization | 0.00 |
| Response to ionizing radiation | 0.00 |
| Cellular response to nutrient levels | 0.00 |
| Protein modification by small protein conjugation | 0.00 |
| Positive regulation of translation | 0.00 |
| Positive regulation of RNA metabolic process | 0.00 |
| Interaction with host | 0.00 |
| Positive regulation of cell cycle | 0.01 |
| Negative regulation of cellular metabolic process | 0.01 |
| Negative regulation of cellular biosynthetic process | 0.01 |
| Positive regulation of cell adhesion | 0.01 |
| Protein polyubiquitination | 0.01 |
| Ras protein signal transduction | 0.01 |
| Protein targeting | 0.01 |
| Nuclear transport | 0.01 |
| Response to hypoxia | 0.01 |
| Negative regulation of signal transduction | 0.01 |
| Response to abiotic stimulus | 0.01 |
| Regulation of protein kinase activity | 0.01 |
| Positive regulation of protein metabolic process | 0.01 |
| Transcription from RNA polymerase II promoter | 0.01 |
| Intracellular protein transport | 0.01 |
| DNA integrity checkpoint | 0.01 |
| Regulation of transferase activity | 0.01 |
| Protein phosphorylation | 0.01 |
| ER-nucleus signaling pathway | 0.01 |
| Organelle localization | 0.01 |
| Protein stabilization | 0.01 |
| Growth | 0.01 |
| Protein export from nucleus | 0.01 |
| Negative regulation of apoptotic process | 0.01 |
| Negative regulation of apoptotic process | 0.01 |
| Positive regulation of protein modification process | 0.01 |
| Positive regulation of binding | 0.01 |
| Negative regulation of biosynthetic process | 0.01 |
| Cellular response to stress | 0.01 |
| Notch signaling pathway | 0.01 |
| Protein modification process | 0.01 |
| Negative regulation of nucleobase-containing compound metabolic process | 0.01 |
| Protein autophosphorylation | 0.01 |
| Cellular response to extracellular stimulus | 0.01 |
| Nucleocytoplasmic transport | 0.01 |
| Phosphorylation | 0.01 |
| Positive regulation of nucleobase-containing compound metabolic process | 0.01 |
| Regulation of sequence-specific DNA binding transcription factor activity | 0.01 |
| Regulation of cell migration | 0.01 |
| Positive regulation of hydrolase activity | 0.01 |
| Regulation of cyclin-dependent protein kinase activity | 0.01 |
| Negative regulation of programmed cell death | 0.01 |
| Establishment of protein localization | 0.01 |
| Negative regulation of growth | 0.01 |
| Regulation of transcription, DNA-dependent | 0.01 |
| Regulation of transcription, DNA-dependent | 0.01 |
| Regulation of transcription, DNA-dependent | 0.01 |
| Positive regulation of catalytic activity | 0.01 |
| Negative regulation of cell migration | 0.01 |
| Small gtpase mediated signal transduction | 0.01 |
| Regulation of gene expression, epigenetic | 0.01 |
| Negative regulation of phosphorylation | 0.01 |
| Phosphatidylinositol-mediated signaling | 0.01 |
| Gene silencing | 0.02 |
| Morphogenesis of an epithelium | 0.02 |
| Regulation of gene expression | 0.02 |
| Viral infectious cycle | 0.02 |
| G2/M transition of mitotic cell cycle | 0.02 |
| Endoplasmic reticulum unfolded protein response | 0.02 |
| Macromolecule modification | 0.02 |
| Regulation of RNA metabolic process | 0.02 |
| Response to radiation | 0.02 |
| Protein transport | 0.02 |
| Regulation of chromosome organization | 0.02 |
| Regulation of viral reproduction | 0.02 |
| Regulation of DNA metabolic process | 0.02 |
| Positive regulation of protein phosphorylation | 0.02 |
| Positive regulation of cell differentiation | 0.02 |
| Regulation of myeloid cell differentiation | 0.02 |
| Cell cycle process | 0.02 |
| Regulation of growth | 0.02 |
| Regulation of cell adhesion | 0.02 |
| Intracellular receptor mediated signaling pathway | 0.02 |
| Positive regulation of developmental process | 0.02 |
| Regulation of mitotic cell cycle | 0.02 |
| Endosomal transport | 0.02 |
| Negative regulation of response to stimulus | 0.02 |
| Positive regulation of cell migration | 0.02 |
| Positive regulation of phosphorylation | 0.02 |
| Mitotic cell cycle | 0.02 |
| Negative regulation of translation | 0.02 |
| Myeloid cell differentiation | 0.02 |
| Viral reproduction | 0.02 |
| Response to UV | 0.02 |
| Protein folding | 0.02 |
| Positive regulation of defense response | 0.02 |
| Cell cycle checkpoint | 0.02 |
| Negative regulation of metabolic process | 0.03 |
| Homeostasis of number of cells | 0.03 |
| Regulation of catalytic activity | 0.03 |
| Positive regulation of cellular metabolic process | 0.03 |
| Response to light stimulus | 0.03 |
| Response to drug | 0.03 |
| Mitochondrial membrane organization | 0.03 |
| Androgen receptor signaling pathway | 0.04 |
| Tissue morphogenesis | 0.04 |
| Stress-activated protein kinase signaling cascade | 0.04 |
| Positive regulation of cysteine-type endopeptidase activity involved in apoptotic process | 0.04 |
| Positive regulation of cellular component organization | 0.04 |
| Positive regulation of transferase activity | 0.04 |
| Cytoskeleton-dependent intracellular transport | 0.05 |
| Regulation of organelle organization | 0.05 |
| Negative regulation of phosphate metabolic process | 0.05 |
| Intracellular transport | 0.05 |
| Regulation of molecular function | 0.05 |
| Positive regulation of I-kappab kinase/NF-kappab cascade | 0.05 |
| Gland development | 0.05 |
| Regulation of cell-cell adhesion | 0.05 |
| Positive regulation of signal transduction | 0.05 |
| Negative regulation of cellular component organization | 0.05 |
| Organelle organization | 0.05 |
| Transmembrane receptor protein serine/threonine kinase signaling pathway | 0.05 |
| Hemostasis | 0.06 |
| Positive regulation of metabolic process | 0.06 |

**Table S4**: Overrepresentation analysis of shared genes between the differentially expressed miRNAs highlights target genes involved in metabolic related pathways.

| GO Categories | P-val |
| --- | --- |
| Mitotic cell cycle | 0.02 |
| Cellular protein catabolic process | 0.02 |
| Interphase | 0.02 |
| Interphase of mitotic cell cycle | 0.02 |
| G1/S transition of mitotic cell cycle | 0.02 |
| Protein catabolic process | 0.03 |
| G1 phase of mitotic cell cycle | 0.03 |
| B cell differentiation | 0.03 |
| Protein modification process | 0.03 |
| G1 phase | 0.03 |
| Macromolecule modification | 0.03 |
| Regulation of cell cycle | 0.04 |
| Apoptotic signaling pathway | 0.04 |
| Small gtpase mediated signal transduction | 0.04 |
| Ras protein signal transduction | 0.04 |
| Regulation of mitotic cell cycle | 0.04 |
| Response to UV | 0.04 |
| Regulation of catabolic process | 0.04 |
| Dephosphorylation | 0.04 |
| Regulation of protein modification process | 0.04 |
| Regulation of protein stability | 0.04 |
| Regulation of cellular protein metabolic process | 0.04 |
| Cellular macromolecule catabolic process | 0.04 |
| Cellular protein metabolic process | 0.04 |
| Positive regulation of cell cycle | 0.04 |
| Regulation of protein phosphorylation | 0.05 |

References

1 Al‑Kafaji G, Al‑Mahroos G, Alsayed N, Hasan Z, Nawaz S, Bakhiet M. Peripheral blood microRNA-15a is a potential biomarker for type 2 diabetes mellitus and pre-diabetes. Mol Med Rep. 2015;**12**:7485–90.

2 Sun L-L, Jiang B-G, Li W-T, Zou J-J, Shi Y-Q, Liu Z-M. MicroRNA-15a positively regulates insulin synthesis by inhibiting uncoupling protein-2 expression. Diabetes Res Clin Pract. 2011;**91**:94–100.

3 Elena Flowers Meghana Gadgil BEA and AMK. Circulating micrornas associated with glycemic impairment and progression in Asian Indians. Proc Natl Acad Sci. 2015;**112**:E2366–75.

4 Zhao C, Popel AS, Rusinova I, Saulep D, Wang D, Xu D. Computational Model of MicroRNA Control of HIF-VEGF Pathway: Insights into the Pathophysiology of Ischemic Vascular Disease and Cancer. PLOS Comput Biol. 2015;**11**:e1004612.

5 Liang X, Xu Z, Yuan M, Zhang Y, Zhao B, Wang J *et al.* MicroRNA-16 suppresses the activation of inflammatory macrophages in atherosclerosis by targeting PDCD4. Int J Mol Med. 2016;**37**:967–75.

6 Lee DE, Brown JL, Rosa ME, Brown LA, Perry RA, Wiggs MP *et al.* MicroRNA-16 Is Downregulated During Insulin Resistance and Controls Skeletal Muscle Protein Accretion. J Cell Biochem. 2016;**117**:1775–87.

7 Chen Y, Tian L, Wan S, Xie Y, Chen X, Ji X *et al.* MicroRNA-17-92 cluster regulates pancreatic beta-cell proliferation and adaptation. Mol Cell Endocrinol. 2016;**437**:213–23.

8 Maha Coucha Islam N Mohamed Sally L Elshaer Osinakachuk Mbata Megan L Bartasis Azza B El-Remessy. High fat diet dysregulates microRNA-17-5p and triggers retinal inflammation: Role of endoplasmic-reticulum-stress - The University of Auckland. World J Diabetes. 2017;**8**:56–65.

9 He QF, Wang LX, Zhong JM, Hu RY, Fang L, Wang H *et al.* Circulating microRNA-21 is downregulated in patients with metabolic syndrome. Biomed Environ Sci. 2016;**29**:385–89.

10 Li S, Fan Q, He S, Tang T, Liao Y, Xie J. MicroRNA-21 Negatively Regulates Treg Cells Through a TGF-�1/Smad-Independent Pathway in Patients with Coronary Heart Disease. Cell Physiol Biochem. 2015;**37**:866–78.

11 Hackett EE, Sheedy FJ. MiR-21 alters circulating Treg function in vascular disease-hope for restoring immunoregulatory responses in atherosclerosis? Ann Transl Med. 2017;**5**:21.

12 Shvangiradze T, Bondarenko I, Troshina E, Shestakova M, Ilyin A, Nikankina L *et al.* Profile of microRNAs associated with coronary heart disease in patients with type 2 diabetes. Obe Metab. 2016;**13**:34.

13 Tang S, Wang F, Shao M, Wang Y, Zhu H. MicroRNA-126 suppresses inflammation in endothelial cells under hyperglycemic condition by targeting HMGB1. Vascul Pharmacol. 2017;**88**:48–55.

14 Rawal S, Munasinghe PE, Shindikar A, Paulin J, Cameron V, Manning P *et al.* Down-regulation of proangiogenic microRNA-126 and microRNA-132 are early modulators of diabetic cardiac microangiopathy. Cardiovasc Res. 2017;**113**:90–101.

15 Pan X, Hou R, Ma A, Wang T, Wu M, Zhu X *et al.* Atorvastatin Upregulates the Expression of miR-126 in Apolipoprotein E-knockout Mice with Carotid Atherosclerotic Plaque. Cell Mol Neurobiol. 2017;**37**:29–36.

16 Witkowski M, Weithauser A, Tabaraie T, Steffens D, Kränkel N, Witkowski M *et al.* MicroRNA 126 Reduces the Blood Thrombogenicity in Diabetes Mellitus via Targeting of Tissue FactorHighlights. Arterioscler Thromb Vasc Biol. 2016;**36**.

17 Al-Kafaji G, Al-Mahroos G, Abdulla Al-Muhtaresh H, Sabry MA, Abdul Razzak R, Salem AH. Circulating endothelium-enriched microRNA-126 as a potential biomarker for coronary artery disease in type 2 diabetes mellitus patients. Biomarkers. 2016;:1–11.

18 Tsukita S, Yamada T, Takahashi K, Munakata Y, Hosaka S, Takahashi H *et al.* MicroRNAs 106b and 222 Improve Hyperglycemia in a Mouse Model of Insulin-Deficient Diabetes via Pancreatic β-Cell Proliferation. EBioMedicine. 2017;**15**:163–72.

19 Nardelli C, Granata I, Iaffaldano L, D ’argenio V, Monaco V Del, Maruotti GM *et al.* MiR-138/miR-222 Overexpression Characterizes the miRNome of Amniotic Mesenchymal Stem Cells in Obesity. doi:10.1089/scd.2016.0127.

20 Feng B, Chakrabarti S. MiR-320 Regulates Glucose-Induced Gene Expression in Diabetes. ISRN Endocrinol. 2012;**2012**:1–6.

21 Chien H-Y, Lee T-P, Chen C-Y, Chiu Y-H, Lin Y-C, Lee L-S *et al.* Circulating microRNA as a diagnostic marker in populations with type 2 diabetes mellitus and diabetic complications. J Chinese Med Assoc. 2015;**78**:204–11.

22 Fomison I, Katare R. Emerging Roles of Micrornas in Diabetic Cardiomyopathy. J Diabetes Metab. 2014;**05**:1–6.

23 Motawae TM, Ismail MF, Shabayek MI, Seleem MM. MicroRNAs 9 and 370 Association with Biochemical Markers in T2D and CAD Complication of T2D. PLoS One. 2015;**10**:e0126957.

24 Liu H, Yang N, Fei Z, Qiu J, Ma D, Liu X *et al.* Analysis of plasma miR-208a and miR-370 expression levels for early diagnosis of coronary artery disease. Biomed reports. 2016;**5**:332–36.

25 Iliopoulos D, Drosatos K, Hiyama Y, Goldberg IJ, Zannis VI. MicroRNA-370 controls the expression of MicroRNA-122 and Cpt1α and affects lipid metabolism. J Lipid Res. 2010;**51**:1513–23.

26 Higuchi C, Nakatsuka A, Eguchi J, Teshigawara S, Kanzaki M, Katayama A *et al.* Identification of Circulating miR-101, miR-375 and miR-802 as Biomarkers for Type 2 Diabetes. Metabolism. 2015;**64**:489–97.

27 Sun K, Chang X, Yin L, Li J, Zhou T, Zhang C *et al.* Expression and DNA methylation status of microRNA-375 in patients with type 2 diabetes mellitus. Mol Med Rep. 2013;**9**:967–72.

28 Yamada H, Suzuki K, Ichino N, Ando Y, Sawada A, Osakabe K *et al.* Associations between circulating microRNAs (miR-21, miR-34a, miR-122 and miR-451) and non-alcoholic fatty liver. Clin Chim Acta. 2013;**424**:99–103.

29 Karimi-Sales E, Jeddi S, Ebrahimi-Kalan A, Alipour MR. Trans-Chalcone prevents insulin resistance and hepatic inflammation and also promotes hepatic cholesterol efflux in high-fat diet-fed rats: modulation of miR-34a-, miR-451-, and miR-33a-related pathways. Food Funct. 2018;**9**:4292–98.

30 Olioso D, Dauriz M, Bacchi E, Negri C, Santi L, Bonora E *et al.* Effects of aerobic and resistance training on circulating micro-RNA expression profile in subjects with type 2 diabetes. J Clin Endocrinol Metab. 2018. doi:10.1210/jc.2018-01820.
